# Assessing the occurrence and severity of pre‐ and postendodontic pain in anemic and healthy female patients

**DOI:** 10.1002/cre2.799

**Published:** 2023-10-17

**Authors:** Maryam Kazemipoor, Hooman Moradi, Fatemeh Mokhtari, Khatereh Kheirollahi

**Affiliations:** ^1^ Department of Endodontics, School of Dentistry Shahid Sadoughi University of Medical Sciences Yazd Iran; ^2^ Department of Oral Medicine, School of Dentistry Shahid Sadoughi University of Medical Sciences Yazd Iran

**Keywords:** iron deficiency anemia, pain, post endodontic, visual analog scale

## Abstract

**Objectives:**

The objective of this current survey was to assess both the occurrence and severity of pre‐ and post‐endodontic pain among female individuals, distinguishing between those with anemia and those without.

**Materials and Methods:**

In the current study, we included a total of 60 women with anemia (hemoglobin < 11) and those without anemia (hemoglobin > 13). We recorded the occurrence and severity of pain before and at 24, 48, and 72 h following root canal treatment using a visual analog scale (VAS) ranging from 0 to 10. Additionally, we collected data on patient age, tooth type, as well as pulpal and periapical conditions. Statistical analysis was carried out using two‐way ANOVA, pairedt‐tests, and Pearson correlation coefficient. The significance level for statistical tests was set at *p* ≤ .05.

**Results:**

The incidence of preoperative pain in both anemic and non‐anemic patients was 80%. The total incidence of post‐operative pain was recorded as 71.7% (82.2% in anemic and 61.1% in non‐anemic women). Compared with the pretreatment pain incidence and intensity, the values increased in 24 h but declined in 48 h and 72 h after treatment. Considering the posttreatment pain intensity, the mean values were higher in the three time intervals (24, 48, and 72 h) in anemic patients. Pulpal and periapical status, in contrast to age and tooth type, significantly contributed to the intensity of posttreatment pain.

**Conclusion:**

Regarding the importance of pain phenomenon in human life, it is recommended to consider anemia as an important risk factor for post‐endodontic pain. Early diagnosis and analgesic treatment interventions in anemic females, alongside the pain control during root canal treatment, could promote the patient's satisfaction and quality of care.

## 1 INTRODUCTION

Dental‐related orofacial pain is the most prevalent form of discomfort in the orofacial region (Hargreaves & Abbott, 2005). The tooth is abundantly supplied with afferent nerve fibers that mainly convey sensations of pain triggered by heat, chemical, and mechanical stimuli (Jain et al., 2013). When these nerve endings within the dental pulp and surrounding tissues are activated by damaging stimuli or the release of inflammatory substances, they transmit nerve signals to the central nervous system, ultimately registering as the sensation of pain (Jain et al., 2013).

Pain is one of the most important complaints of dental patients, and rationale treatment generally involves removing the causes of pain (Saatchi et al., 2011).

Various factors such as age, sex, and type of tooth (Ryan et al., 2008), pretreatment pain (Arias et al., 2009), premature contact in temporary repair (Saatchi & Mansouri, 2005), change in periapical tissue pressure (Seltzer, 2004), factors during treatment, number of treatment sessions (El Mubarak et al., 2010), immunological factors, and changes in cyclic nucleotides (Seltzer & Naidorf, 1985), psychological issues (Oshima et al., 2009), microbial factors (Siqueira, 2003), systemic diseases like anemia (Kawar et al., 2018), and chemical mediators (Jain et al., 2013) are effective in causing these types of pain.

According to studies, patients with sickle cell anemia experience severe pain during periods of vascular obstruction, including odontogenic pain (Kawar et al., 2018). Red blood cells containing sickle hemoglobin undergo a transformation, becoming rigid and causing difficulty in passing through narrow blood vessels. This obstruction impedes or slows down blood circulation, resulting in prolonged microvascular blockage and subsequent oxygen deficiency, which can ultimately lead to tissue ischemia (Ballas et al., 2012; Kawar et al., 2018). Hypoxia, in turn, gives rise to systemic complications like local vascular constriction and episodes of pain that can inflict harm on essential organs (Ballas et al., 2012). Within the oral cavity, this condition can lead to ischemia and tissue death in facial bones, even causing osteomyelitis unrelated to dental issues, particularly in the mandible (Al‐Jafar et al., 2016; Ballas et al., 2012). Furthermore, research has indicated that the blockage of microvasculature in dental pulp, even in healthy teeth, may trigger pulpal inflammation (Ballas et al., 2012) and potentially result in pulpal necrosis (Al‐Jafar et al., 2016). When the pulp becomes inflamed, changes in tissue pressure, stemming from reduced blood flow and oxygen levels, can affect nerve‐free endings, leading to damage and stimulation of pulp nerves (Jain et al., 2013; Siqueira, 2003; Souza et al., 2017).

Also, studies have shown that chronic ischemic pain can also occur in peripheral arterial disease (Rüger et al., 2008), and blood vessel dysfunction itself may be a potential mechanism of pain (Lim et al., 2015).

Iron deficiency anemia has been documented to be prevalent among females in Iran, with a reported rate of 52.3% (Fesharakiniya et al., 2007). Given the absence of previous research exploring the connection between iron deficiency anemia and the occurrence and intensity of pain following root canal treatment, the objective of this study is to examine and compare the incidence and severity of post‐root canal treatment pain among women both with and without iron deficiency anemia.

## 2 MATERIALS AND METHODS

All experimental procedures conducted in this study received approval from the Ethical Committee of Research at Shahid Sadoughi University of Medical Sciences in Yazd (IR.SSU.REC.1397.106). Before commencing the study, informed consent was obtained from each patient. This descriptive cross‐sectional study involved the participation of 60 female patients (*n* = 60) aged 18–54 years. These patients had previously sought treatment at a specialized clinic in Yazd and exhibited clinical diagnoses including irreversible pulpitis with or without acute periapical periodontitis, pulp necrosis with acute periapical periodontitis, and pulp necrosis with acute periapical abscess in their lower posterior teeth, all of which were included in the study. According to the study's design, 30 women with hemoglobin levels above 13 (indicative of no iron deficiency anemia) and 30 women with hemoglobin levels below 11 (indicative of iron deficiency anemia) participated. Patients with a history of referred pain from maxillary teeth, any underlying systemic diseases or medications other than those related to iron deficiency anemia, the use of different doses and types of analgesics not prescribed in the study during treatment and follow‐up, diagnosis of procedural errors, unsuccessful anesthesia, or the need for a second injection were excluded.

An endodontist performed endodontic treatment for all patients under the same conditions. Pretreatment pain in visual analog scale (VAS) scale was recorded for every patient. Standard procedure for root canal therapy includes IAN injections with one cartridge of 2% lidocaine with 1:80,000 epinephrine, rubber dam isolation, caries excavation, access cavity preparation, cleansing and shaping, and finally obturation for each patient. Instrumentation of the root canal was completed using hand or rotary‐based endodontic system ProTaper universal files (ProTaper Universal, Dentsply Maillefer). 5.25% Sodium hypochlorite solution was used for intracanal irrigation, and the final irrigation was performed with normal saline. Root canals were obturated with AH‐26 root canal sealer (Dentsply) and gutta‐percha using the lateral condensation technique.

About 400 mg of ibuprofen (Gelofen 400) every 8 h (three doses) was administered to all patients for pain control after root canal therapy. 24, 48, and 72 h after endodontic treatment, the presence or absence of pain or pain severity was recorded according to the VAS scale.

Data were analyzed using SPSS software (ver. 17), two‐way analysis of variance, paired *t* test, and Pearson correlation coefficient.

## 3 RESULTS

**Figure 1:**
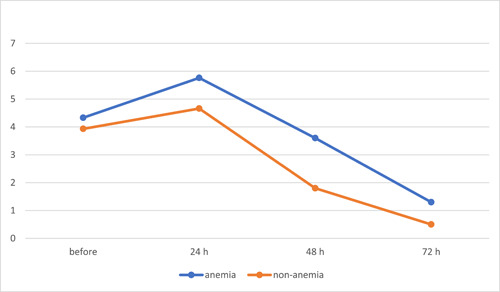
Changes in the visual analog scale for pain score of the two groups of patients studied. Group A (blue) = anemic patients; Group B (red) = non‐anemic patients.

In this descriptive cross‐sectional study, 60 female patients participated, with an average age of 33.75 ± 10.29 and an age range spanning from 18 to 54 years. Among these patients, 30 were diagnosed with iron deficiency anemia (Hb < 11), while the remaining 30 did not exhibit iron deficiency anemia (Hb > 13). The mean hemoglobin level for the entire study group was 11.86 ± 2.15, ranging between 7.70 and 14.90. In the group with anemia, the mean hemoglobin level averaged 9.83 ± 0.82, ranging from 7.7 to 11, while the non‐anemic group had a mean hemoglobin level of 13.88 ± 0.57, ranging from 13.01 to 14.90. Distribution of the tooth type in patients was 16.9% mandibular first premolars, 27.1% mandibular second premolars, 35.6% mandibular first molars, and 20.3% mandibular second molars. Based on pulpal status, 36.7% of patients had necrotic teeth, and 63.3% had irreversible pulpitis. Based on periapical status, 41.7% of patients had acute periapical periodontitis, 1.7% had acute periapical abscess, and 56.7% had normal periapical status. Generally, the incidence of preoperative pain in both groups with iron deficiency anemia and without iron deficiency anemia was 80%. The incidence of pain after treatment was generally 71.66% (82.23% in people with iron deficiency anemia and 61.12% in people without iron deficiency anemia). In patients with anemia, the incidence of pain after treatment at 24, 48, and 72 h was reported to be 100%, 96.67%, and 50%, respectively. In patients without anemia, the incidence of pain after treatment at 24, 48, and 72 h was 100%, 53.34%, and 30%, respectively. The mean pain scores in the two groups and the four time intervals, before treatment and 24, 48, and 72 h after treatment, are given in Table 1 and Figure 1. Based on the *t* test in comparison to anemic and non‐anemic patients, there were no significant differences in preoperative pain (*p*‐value = .643) and postoperative pain at 24 h (*p*‐value = .093).

**Table 1:** Comparison of preoperative and postoperative pain in anemic and non‐anemic patients (results are expressed as mean ± SD).

Pain (VAS) groups	*n*	Before	24 h	48 h	72 h
Anemia	30	4.33 ± 3.41	5.76 ± 1.95	3.60 ± 2.20	1.30 ± 1.80
Non‐anemia	30	3.93 ± 3.22	4.66 ± 2.92	1.80 ± 2.41	0.50 ± 0.90
Total	60	4.13 ± 3.30	5.21 ± 2.53	2.70 ± 2.46	0.90 ± 1.46

Abbreviation: VAS, visual analog scale.

In contrast, based on the *t* test in comparison to anemic and non‐anemic patients, there were significant differences in postoperative pain at 48 h (*p*‐value = .004) and 72 h (*p*‐value = .034).

The results showed that the mean difference between increasing pain before treatment and 24 h after treatment in the group of people without iron deficiency anemia was −0.73. There was no statistically significant difference between pain before treatment and 24 h after treatment in people without iron deficiency anemia (*p*‐value = .118). The mean difference between increasing pain before treatment and 24 h after treatment in the group of people with iron deficiency anemia was −1.43. In terms of increased pain, there was a statistically significant difference between pain before treatment and pain 24 h after treatment in people with iron deficiency anemia (*p* = .007).

The results showed that the mean difference in pain reduction before treatment and 48 h after treatment in the group without iron deficiency anemia was 2.13. That is, in terms of pain reduction, there was a statistically significant difference between pain before treatment and pain 48 h after treatment in people without iron deficiency anemia (*p*‐value = .003). Based on these results, the mean difference in pain reduction before treatment and 48 h after treatment in the group of people with iron deficiency anemia was 0.73. There was no statistically significant difference between the pain before treatment and 48 h after treatment in people with iron deficiency anemia (*p*‐value = .163).

The results showed that the mean difference in pain reduction before treatment and 72 h after treatment in the group of people without iron deficiency anemia was 3.43. In terms of pain reduction, there was a statistically significant difference between pain before treatment and pain 72 h after treatment in people without iron deficiency anemia (*p*‐value = .000). The mean difference in pain reduction before treatment and 72 h after treatment in the group of people with iron deficiency anemia was 3.03. In other words, there was a statistically significant difference between pain before treatment and pain 72 h after treatment in people with iron deficiency anemia (*p*‐value = .000).

The results showed that patients' age in the four time periods had no significant effect on the mean pain score reported in patients. Regarding the type of teeth in the four time periods, there was no significant difference in the mean pain score reported by patients.

Based on the results, the condition of the pulp (vitality and necrosis) in the mandibular premolars and molars in three time periods had a significant effect on the mean difference between preoperative and postoperative pain scores reported in patients.

In necrotic teeth, higher pain was reported at 24 h after treatment compared to teeth with irreversible pulpitis. Over the periods of 48 and 72 h after treatment, in both necrotic and irreversible pulpitis teeth, a decrease in pain was observed, but teeth with irreversible pulpitis showed a greater decrease in the reported mean pain score.

Considering the periapical status, only 72 h after treatment, there was a significant difference in the mean pain score between pre‐post treatment pains reported in patients. At 24 h after treatment, an increase in pain was observed in both teeth with acute periapical periodontitis and normal teeth, but this increase was greater in normal teeth. Pain reduction was observed within 48 h in both groups, and this reduction was more in patients with acute periapical periodontitis. At 72 h after treatment, teeth with acute periapical periodontitis showed a significant reduction in pain compared to normal teeth.

As per the Pearson correlation analysis, within the non‐anemic group, there existed a noteworthy and positive correlation between pain levels before treatment and pain experienced 24 h posttreatment. Additionally, there was a positive but not statistically significant correlation between pain before treatment and the pain reported at 48 and 72 h following treatment. In contrast, in the anemic group, there was a significant and positive correlation between pain before treatment and the pain experienced at 24, 48, and 72 h posttreatment. Furthermore, within the non‐anemic group, significant positive correlations were observed between pain levels at 24 and 48 h, 24 and 72 h, and 48 and 72 h after treatment. Similarly, in the anemia group, there were positive and significant correlations between pain levels at 24 and 48 h, 24 and 72 h, and 48 and 72 h after treatment.

## 4 DISCUSSION

Odontogenic pain following root canal treatment is a great concern for both clinicians and patients. The ability to predict the pain prevalence after endodontic treatment and its predisposing factors help the clinician to better control the treatment procedures, which promotes the patient's confidence in the clinician (Ng et al., 2004).

Persistent pain (>1‐month pain duration) after root canal therapy has been reported in the previous surveys from 3% to 12% (Nixdorf et al., 2016; Polycarpou et al., 2005). Incidence of post‐endodontic pain (<1‐month pain duration) also has been widely reviewed, but the recordings showed a high variability ranging from 82.9% to 10.6% (Polycarpou et al., 2005).

Postoperative pain can be influenced by various factors, categorized as patient‐related, tooth‐related, and clinician‐related (Nixdorf et al., 2016). It is well established that the physical and mental condition of patients plays a significant role in the occurrence of postendodontic pain (Ng et al., 2004).

Surprisingly, the incidence of postendodontic pain in relation to iron deficiency anemia has not been studied until now. The primary objective of this study was to evaluate both the incidence and intensity of pain in patients with and without iron deficiency anemia undergoing root canal treatment. The overall incidence of pain before treatment was noted at 80% for both anemic and non‐anemic patients. After treatment, the overall incidence of pain was 71.7%, with 82.2% in anemic patients and 61.1% in non‐anemic patients. It is worth noting that using the VAS scale, and most studies tend to report 100% prevalence because even the slightest discomfort registers as a pain score greater than 0.

In this study, compared to the pain experienced before root canal therapy, the incidence of pain increased at 24 h but decreased at 48 and 72 h after the procedure. Ng et al. (2004) found that the prevalence of postobturation pain can vary widely, from 0% (at 30 days) to 65% (at 1 day). The prevalence of postobturation pain within 48 h after treatment was 40.2% but decreased over time.

While many studies have explored the incidence and prevalence of posttreatment pain, they have typically been either randomized controlled trials (Koba et al., 1999) or prospective studies (Albashaireh & Alnegrish, 1998; Fava, 1991). However, variations in inclusion criteria, treatment approaches, time frames for pain assessment, pain measurement methods, and clinician experience have made comparing these studies quite challenging. Pak and White (2011), in a systematic review, evaluated pain prevalence and severity before, during, and after root canal treatment. The results revealed that the prevalence of pretreatment endodontic pain was high (28%) but dropped moderately within 1 day (24%) and reached the minimal level in 7 days (14%). Also, the severity of posttreatment endodontic pain was moderate but decreased within 1 day following treatment and reached a minimal level in 7 days.

Factors associated with posttreatment pain include gender (Genet et al., 1987), tooth type or location (Yesilsoy et al., 1988), the presence and intensity of pretreatment pain (Albashaireh & Alnegrish, 1998; Yesilsoy et al., 1988), pulpal status (Albashaireh & Alnegrish, 1998; Genet et al., 1987), presence and size of periapical lesion (Genet et al., 1987), intra canal irrigation and medicament (Harrison et al., 1983), number of treatment visits (Adeyemo et al., 2011; Yesilsoy et al., 1988), extent of root canal obturation (Yesilsoy et al., 1988), and number of root canals (Genet et al., 1987).

The incidence of chronic pain in women was found to be approximately four times higher compared with men. Also, considering the prevalence of postobturation pain, there were significant differences between men and women (Genet et al., 1987; Ng et al., 2004).

Various explanations that have been presented with regard to higher prevalence of posttreatment pain in women included psychosomatic status, emotional factors, and biological differences (Ng et al., 2004). Reproductive organs in women may provide an additional portal of infection entry that leads to possible local and distant hyperalgesia. Moreover, fluctuating female hormonal levels may trigger the production of serotonin and noradrenaline, which affects the pain prevalence in the menstrual period and women receiving hormone replacement therapy or oral contraceptives (Dao et al., 1998).

Numerous factors are associated with posttreatment pain, categorized as patient‐related, tooth‐related, and clinician‐related (Nixdorf et al., 2016). Iron deficiency anemia, resulting from insufficient dietary iron or chronic blood loss, is the most common hematological disorder (Berkley, 1997). Approximately 50% of anemia cases are considered to be due to iron deficiency, but based on population groups and different areas, the recorded proportions vary (Stoltzfus, 2003). Since the prevalence of iron deficiency anemia is significantly higher in women compared to men, it could contribute to the increased incidence of postendodontic pain in women (Sinclair & Hinton, 2005).

Iron deficiency anemia hampers the transportation of oxygen by hemoglobin to peripheral tissues, impacting neuronal function (Nieber, 1999). Neurons are sensitive to oxygen availability, and hypoxia triggers various physiological and biochemical responses, including neural hyperexcitability and disturbances in ion concentrations (Nieber, 1999). Hypoxia modified synaptic interaction and disturbed hemostasis, characterized by enhanced cellular K+ efflux and Na+ and Ca2+ influx followed by extracellular acidosis (Tombaugh & Sapolsky, 1993). Depolarization occurs shortly after hypoxia and induces neural hyperexcitability (Nieber, 1999). Higher concentrations of Ca2+ in cytosol may overstimulate Ca2+‐dependent protease, phospholipase, and endonuclease (Epstein et al., 1994). Hypoxia also induces the release of transmitters and changes in membrane potential, which leads to a reversible loss of neuronal function (Nieber, 1999).

Normal functioning of the nervous system also depends on the barrier effect of the neural vasculature, which is mainly attributed to the presence of complex tight junction between endothelial cells (Koto et al., 2007). Claudine 5, a key molecule in the tight junction assembly, was closely correlated with the increase in the transendothelial electrical resistance. Hypoxia altered the location of the protein in the plasma membrane, which resulted in a decrease in the transendothelial electrical resistance (Nieber, 1999). In this study, it was observed that the mean pain intensity before and after treatment was higher in anemic women compared to non‐anemic ones, with significant differences noted after 48 and 72 h. However, while the mean pain score after 72 h may show statistical significance between anemic and non‐anemic patients, it may not hold clinical significance.

The pattern of pain intensity changes was similar in both experimental groups: an initial increase at 24 h posttreatment followed by a decline at 48 and 72 h after treatment.

The results are similar to the results of Albashaireh et al. (1998) and Genet et al. (1987) studies.

Regarding the comparison between pain intensity before and after treatment, a significant increase was observed in anemic patients at 24 h, a significant decrease was observed in non‐anemic women at 48 h, and significant decreases were observed in both anemic and non‐anemic patients at 72 h.

Despite rare surveys directly comparing pretreatment, treatment, and posttreatment pain (Genet et al., 1987; Nixdorf et al., 2016), it appears that the overall pattern of pain prevalence and severity follows a consistent trend: a decrease over time. However, in this study, the increase in pain intensity at 24 h after treatment, especially in anemic patients, diverged from this pattern.

In contrast to some earlier studies (Ng et al., 2004; Pak & White, 2011; Polycarpou et al., 2005), this study's findings suggest that the increase in pain intensity 24 h after treatment may be related to sample characteristics and the method used to measure pain intensity. The prevalence and intensity of pain were higher in women compared to men, which could explain the difference in pain intensity trends observed at 24 h posttreatment.

While Polycarpou et al. (2005) found that the intensity of preoperative pain did not significantly influence the prevalence of chronic pain after root canal therapy, other studies have documented a link between pretreatment pain intensity and posttreatment flare‐ups (Jain et al., 2013; Pak & White, 2011).

In non‐anemic patients, the present study revealed a significant positive correlation between pain intensity at 24 h and pretreatment pain. However, in anemic patients, there was a significant positive correlation between pain intensity at all three posttreatment time intervals and pretreatment pain.

The heightened pain intensity observed within the first day following root canal treatment may be attributed to ongoing inflammatory processes, particularly when periradicular inflammation is present, as well as neural hypersensitivity in periapical regions (Pak & White, 2011).

Research has indicated that patients with a history of previous chronic pain problems, such as somatic pain in anemic patients, were approximately 4.5 times more susceptible to orofacial pain despite successful root canal treatment (Polycarpou et al., 2005). This finding aligns with the present study, which reported higher pain intensity and incidence following surgery in anemic patients.

While Ng et al. (2004) found that molar teeth were significantly more prone to postendodontic pain due to their complex root canal morphology, in the present study, tooth type (mandibular premolars and molars) did not significantly affect posttreatment pain intensity. This could be due to the higher proportion of molar teeth in the study, as well as the evaluation of both maxillary and mandibular teeth, which may explain the differences between Ng et al. (2004) and the current findings.

In this study, both teeth with acute periapical periodontitis and those with a normal periodontium exhibited an increase in pain intensity at 24 h posttreatment, although the increase was more pronounced in normal teeth. At 48 and 72 h after treatment, both groups showed a statistically significant decrease in pain intensity, with a more pronounced decline in patients with acute periapical periodontitis. It is speculated that cleaning and shaping procedures removed irritants triggering the inflammatory process, and the root canal space might act as a “buffer” against the pressure caused by inflammatory exudate in the periodontal space (Ng et al., 2004).

Similar to the findings of Albashaireh and Alnegrish (1998) and Genet et al. (1987), in the present study, pain intensity and incidence significantly increased in necrotic teeth compared to vital teeth at 24 h after treatment. In vital teeth, the primary source of clinical pain, the inflamed pulp, is removed during root canal therapy. In non‐vital teeth, clinical pain primarily originates in the periapical region, which cannot be completely eliminated during cleaning and shaping. Moreover, over‐instrumentation in necrotic teeth may push infected pulp tissue toward the periapical region, triggering an inflammatory process (Genet et al., 1987). Another contributing factor to posttreatment pain was the root canal preparation method (Arias et al., 2015). Studies have shown that root canal preparation using Protaper NiTi rotary instruments resulted in a lower incidence of posttreatment pain compared to manual preparation, although rotary instrumentation may lead to longer‐lasting pain in the presence of postendodontic pain (Arias et al., 2015). The relatively high percentage of pain incidence at 72 h after treatment in this study (50% in anemic and 30% in non‐anemic patients) could be related to the root canal preparation technique employed here.

It is worth noting that all variables related to the technique and operator were controlled in this research, with all root canal treatments performed by one operator using one technique. Studies have suggested that when intra‐canal medicament is not used, there is no significant difference in the prevalence of postoperative pain (Ng et al., 2004). In this study, all treatment procedures were completed in a single session. As a first step, future research should address the limitations of this study, including sample size, the lack of standardization of teeth (premolars and molars with different success rates), and the lack of standardization of initial pulp status, to achieve greater clinical relevance.

## 5 CONCLUSION

Based on the findings of this study, it is evident that postendodontic pain tends to increase within the first 24 h after treatment and then gradually subsides in the subsequent days. Consequently, effective pain management both before and during the initial 24 h following root canal therapy is of utmost importance, particularly for ensuring the comfort and well‐being of female patients during their treatment.

Anemic patients, when compared to their non‐anemic counterparts, exhibited a higher incidence and intensity of pain after undergoing root canal therapy. The notable difference in pain intensity between anemic and non‐anemic patients, notably observed at the 48 and 72 h following treatment, suggests the potential for delayed pain experiences in anemic patients. Therefore, it is imperative to provide additional care and attention to control and prevent postoperative pain in anemic patients and to reduce the risk of central neuroplasticity.

## 6 RECOMMENDATIONS

Given the significance of pain in the human experience and its potential to influence dentists, the public, and other healthcare professionals' perceptions regarding the preservation of natural teeth, it is advisable to recognize anemia as a noteworthy risk factor for the development of postendodontic pain. Timely identification and the implementation of analgesic treatments in anemic females, coupled with effective pain management during root canal procedures, can enhance patient satisfaction and the overall quality of care. Additionally, future interventional studies should be devised to monitor pain incidence in anemic patients throughout the process of anemia treatment, aiming to restore their hemoglobin levels to normal.

## AUTHOR CONTRIBUTIONS

Maryam Kazemipoor developed the initial concept of study. Maryam Kazemipoor, Hooman Moradi, and Fatemeh Mokhtari contributed to the study design and wrote the manuscript. Khatereh Kheirollahi collected the data and Fatemeh Mokhtari oversaw the data collection. Maryam Kazemipoor contributed to the methodology, analysis, and interpretation of data. Maryam Kazemipoor and Khatereh Kheirollahi contributed to the data analysis. All authors revised and approved the final manuscript.

## CONFLICT OF INTEREST STATEMENT

The authors declare no conflict of interest.

## Data Availability

Data available on request from the authors.
